# Systematic Review of Rare Major Adverse Cardiovascular Events Associated With the Treatment of Acne With Isotretinoin

**DOI:** 10.1111/ajd.14424

**Published:** 2025-02-10

**Authors:** Timothy L. Cowan, Madeleine Stark, Simona Sarmiento, Andrew Miller

**Affiliations:** ^1^ The Canberra Hospital Garran Australian Capital Territory Australia; ^2^ School of Medicine and Psychology Australian National University Garran Australian Capital Territory Australia

**Keywords:** acne vulgaris, cardiovascular system, isotretinoin

## Abstract

Acne vulgaris is a common inflammatory condition that is often treated by dermatologists with isotretinoin. Isotretinoin has a number of effects on inflammatory pathways, with clear efficacy in managing acne vulgaris. There are also well‐documented side effects of isotretinoin, including hyperlipidemia; however, the overall cardiovascular risk is unclear. This systematic review revealed low evidence for major adverse cardiovascular events associated with the use of isotretinoin in acne patients. Despite this low evidence, rare events may be associated and should still be considered when using isotretinoin in acne patients.

## 1 Introduction

Acne vulgaris is a common chronic inflammatory skin condition that primarily emerges during puberty and decreases with age [1, 2]. The pathophysiology of acne is complex and involves a range of mechanisms relating to abnormal sebaceous gland growth, stimulation of sebum secretion by androgens, keratinocyte amplification and disruption to the commensal bacterial microflora of the skin with colonisation of the pilosebaceous unit by *Cutibacterium acnes* (*C. acnes*) [3]. Isotretinoin (13‐cis‐retinoic acid) is an effective treatment of acne vulgaris through various pathways leading to decreased numbers of comedones, inflammation, sebum production and *C. acnes* [4]. The ongoing use of isotretinoin as a treatment for moderate to severe acne vulgaris is a testament to the medication's efficacy despite the well‐documented adverse effects such as teratogenicity, mood changes, liver enzyme derangement, xerosis, cheilitis, dry eyes, arthralgia, myalgia and hyperlipidaemia [4]. In addition to the well‐documented phenomenon of isotretinoin‐induced hyperlipidaemia, isotretinoin's effect on nuclear transcription factors results in a cascade of changes to inflammatory pathways through various interleukins resulting in modified inflammation. This can underlie severe initial inflammatory responses including acne fulminans and could be theorised to contribute to an increased cardiovascular risk in those taking isotretinoin [5].

The population of patients taking isotretinoin for management of acne is typically young and otherwise healthy. The risk of isotretinoin‐induced hyperlipidaemia and inflammation in this young and healthy population group is well documented, but any resulting increased risk of major adverse cardiovascular events (MACE) and associated sequelae is unclear. To clarify this risk, we conducted a systematic review of cardiovascular events in acne patients taking isotretinoin.

## 2 Methods

### 2.1 Literature Search

Literature searches were conducted on PubMed, Medline (Ovid), Embase (Ovid) and Scopus with hand searches of the reference lists of included studies. Studies were included if published in English and if it described cardiovascular events in a patient taking isotretinoin for acne. All study designs were included in order to capture rare events. Search terms included in Appendix A focused on synonyms of isotretinoin and aimed to capture various forms of MACE, including events pertaining to ischaemia, arrhythmia, vasculitis, thromboembolism and cardiomyopathy, as well as general cardiovascular descriptors.

### 2.2 Screening and Data Collection

Studies were imported to Covidence with the removal of duplicates. All articles were screened independently by two authors, and conflicts resolved with discussion. A third author was consulted to resolve any remaining conflicts. Extracted data included demographics, details regarding dose, duration and timeline of isotretinoin exposure, comorbidities, cardiovascular events and details regarding comparator groups.

### 2.3 Quality Assessment

Case studies were assessed using the 8‐point JBI case study appraisal checklist [6]. Quality was appraised as ‘good’ with > 7 points, ‘moderate’ for 5–7 points and ‘poor’ if below 5 points. Case series were assessed using the JBI case series appraisal checklist. Non‐randomised cohort studies were appraised using the 8‐point Newcastle‐Ottawa scale with determination of ‘good’, ‘fair’ or ‘poor’ according to the scoring guidelines [7].

### 2.4 Causality Assessment

For case reports and case series, the probability of causality of MACE from isotretinoin exposure was assessed by the 10‐point Naranjo Adverse Drug Reaction Probability scale [8]. Reactions were deemed ‘definite’ in causality from isotretinoin if over 9 points, ‘probable’ if 5–8 points, ‘possible’ if 1–4 points and ‘doubtful’ if 0 points.

### 2.5 Statistical Analyses

Studies with isotretinoin‐exposed and control groups were combined for meta‐analysis, and case studies were reported by a subgroup of cardiovascular events. This study followed PRISMA guidelines and was prospectively registered on PROSPERO [9, 10].

## 3 Results

### 3.1 Literature Search

**FIGURE 1:**
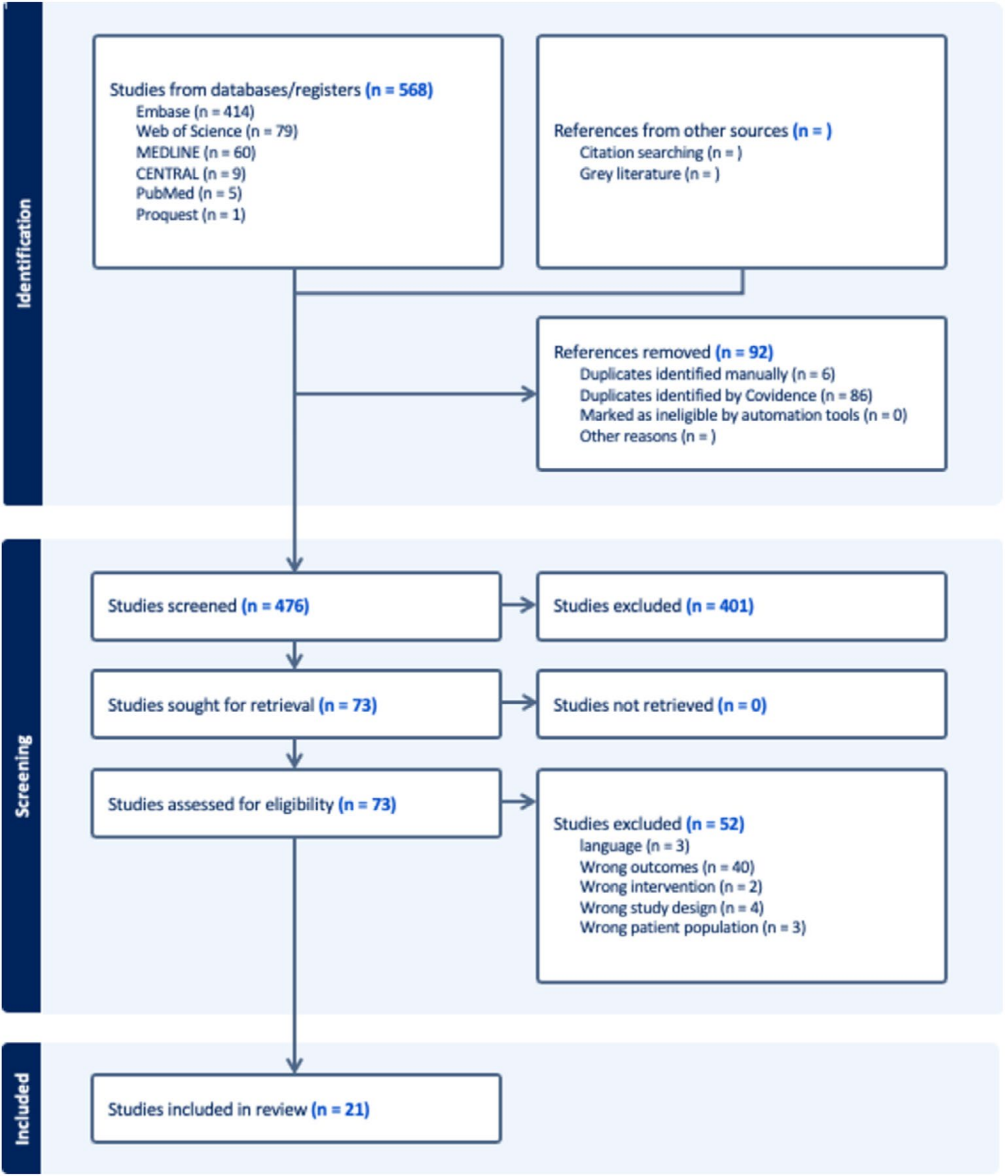
PRISMA flowsheet of literature review. Studies were excluded during screening if patients did not have acne, patients were not treated with isotretinoin, studies were review articles and did not report on new clinical cardiovascular events.

A total of 568 studies were identified. After the removal of duplicates, 476 abstracts were screened and 74 studies underwent full‐text review (Figure 1). Of these, 21 studies were included in the final analysis: 19 were case studies and 2 were non‐randomised retrospective cohort reviews (Table 1). Of these two cohort reviews, one included matched controls and the other had no control group.

**TABLE 1:** Summary of publications included in systematic review. *Average age, **Gender and comorbidities matched between groups.

#	Lead author	Year	Country	Study design	Pt No.	Control	Age	Sex	Comorbidities	Duration of Rx	Daily dose	Cumulative dose	Category (with control)	CV event (with control)	Level of evidence	JBI/NOS quality	Naranjo score	Naranjo probability
1	Agrawal [11]	2015	India	Case report	1	—	22	M	Unreported	2 days	20 mg	40 mg	Thromboembolism	PE	IV	Moderate	4	Possible
2	Akcay [12]	2019	Turkiye	Case report	1	—	25	M	Smoking	7 days	20 mg	140 mg	ACS, thromboembolism	Non‐STEMI due to coronary thrombus	IV	Good	3	Possible
3	Alan [13]	2016	Brazil	Case report	1	—	35	F	Unreported	1 month	30 mg	900 mg	Arrhythmia	Premature ventricular contraction	IV	Good	3	Possible
4	Alatas [14]	2020	USA	Case report	1	—	15	F	Nil	1 day	20 mg	20 mg	ACS	Allergic myocardial infarction	IV	Good	3	Possible
5	Amati [15]	2022	Italy	Case report	1	—	18	M	Nil	Unclear	40 mg	—	Thromboembolism	Renal infarction due to thrombus with left ventricular heart failure	IV	Moderate	3	Possible
6	Annangi [16]	2019	USA	Case report	1	—	21	F	Unreported	2 months	60 mg	3600 mg	Vasculitis	GPA vasculitis with pulmonary renal syndrome	IV	Good	3	Possible
7	Assuncao [17]	2018	Brazil	Case report	1	—	27	M	Nil	3 months	—	—	Vasculitis	GPA vasculitis with diffuse alveolar haemorrhage	IV	Good	3	Possible
8	Charalabopoulos [18]	2003	Greece	Case report	1	—	18	M	Nil	3 months	1 mg/kg	—	Arrhythmia	Right bundle branch block	IV	Good	3	Possible
9	Guler [19]	2014	Turkiye	Case report	1	—	26	F	Nil	4 months	0.5 mg/kg	—	Arrhythmia	Atrial tachycardia with pericardial effusion	IV	Moderate	3	Possible
10	Hasdemir [20]	2005	Turkiye	Case report	1	—	16	M	Nil	3 months	30–40 mg	3300 mg	Arrhythmia	Atrial tachycardia	IV	Good	3	Possible
11	Kilic [21]	2009	Turkiye	Case report	1	—	17	M	Nil	6 months	30 mg	5400 mg	Arrhythmia	Premature ventricular contractions	IV	Good	3	Possible
12	Labiris [22]	2009	Greece	Case report	1	—	17	M	Carrier of prothrombin gene mutation	6 weeks	60 mg	2520 mg	Thromboembolism	Central retinal vein occlusion	IV	Good	3	Possible
13	Laroche [23]	2007	USA	Case report	1	—	30	M	Nil	3 months	45 mg	3780 mg	Thromboembolism	Recurrent cerebral ischaemia	IV	Good	5	Probable
14	Lorenzo [24]	2015	Spain	Case report	1	—	28	F	OCP	12 months	—	—	ACS, thromboembolism	STEMI from RCA plaque and occlusive thrombus	IV	Good	3	Possible
15	Malone [25]	2018	USA	Case report	1	—	14	M	Nil	6 weeks	—	—	Vasculitis	ANCA granulomatosis with polyangiitis affecting lung and kidney	IV	Good	3	Possible
16	Okuyucu [26]	2015	Turkiye	Case report	1	—	27	F	Unreported	2 months	—	—	Thromboembolism	Central venous thrombosis	IV	Good	3	Possible
17	Pepe [27]	2023	Italy	Case report	1	—	18	M	Nil	5 months	—	—	Cardiomyopathy, thromboembolism	Dilated cardiomyopathy and renal thrombosis	IV	Moderate	3	Possible
18	Rashid [28]	2021	USA	Case report	1	—	31	M	Nil	—	—	—	ACS, thromboembolism	Acute coronary syndrome	IV	Poor	3	Possible
19	Reynolds [29]	1989	UK	Case report	1	—	18	M	Nil	6 months	1 mg/kg		Vasculitis	Necrotising small artery vasculitis within muscle	IV	Moderate	3	Possible
20	Rademaker [30]	2010	New Zealand	Retrospective cohort	1743	0	—	—	—	—	—	—	Cardiomyopathy	Cardiomyopathy	IV	Poor	—	—
21	Ghanshani [31]	2021	USA	Retrospective cohort	11,942	11,942	25.9*	**	**	—	—	—	ACS (5), heart failure (4), arrhythmia (6)	Myocardial infarction 5 (8), heart failure 4 (12), AF 3 (9), VT 3 (4)	III	Good	—	—

### 3.2 Quality of Evidence

Of the 19 case studies, 13 were appraised as ‘good’ quality, 5 were ‘moderate’ and 1 was ‘poor’. One cohort study was deemed ‘poor’ quality, and the other, with a matched control group, was ‘good’.

### 3.3 Probability of Causality

The causality of MACE occurring from isotretinoin exposure was deemed ‘possible’ in 18 of the 19 case studies and ‘probable’ in 1 of the 19 studies. No case studies had ‘definite’ causality. Causality was unable to be interpreted from the cohort studies.

### 3.4 MACE Subtypes

**FIGURE 2:**
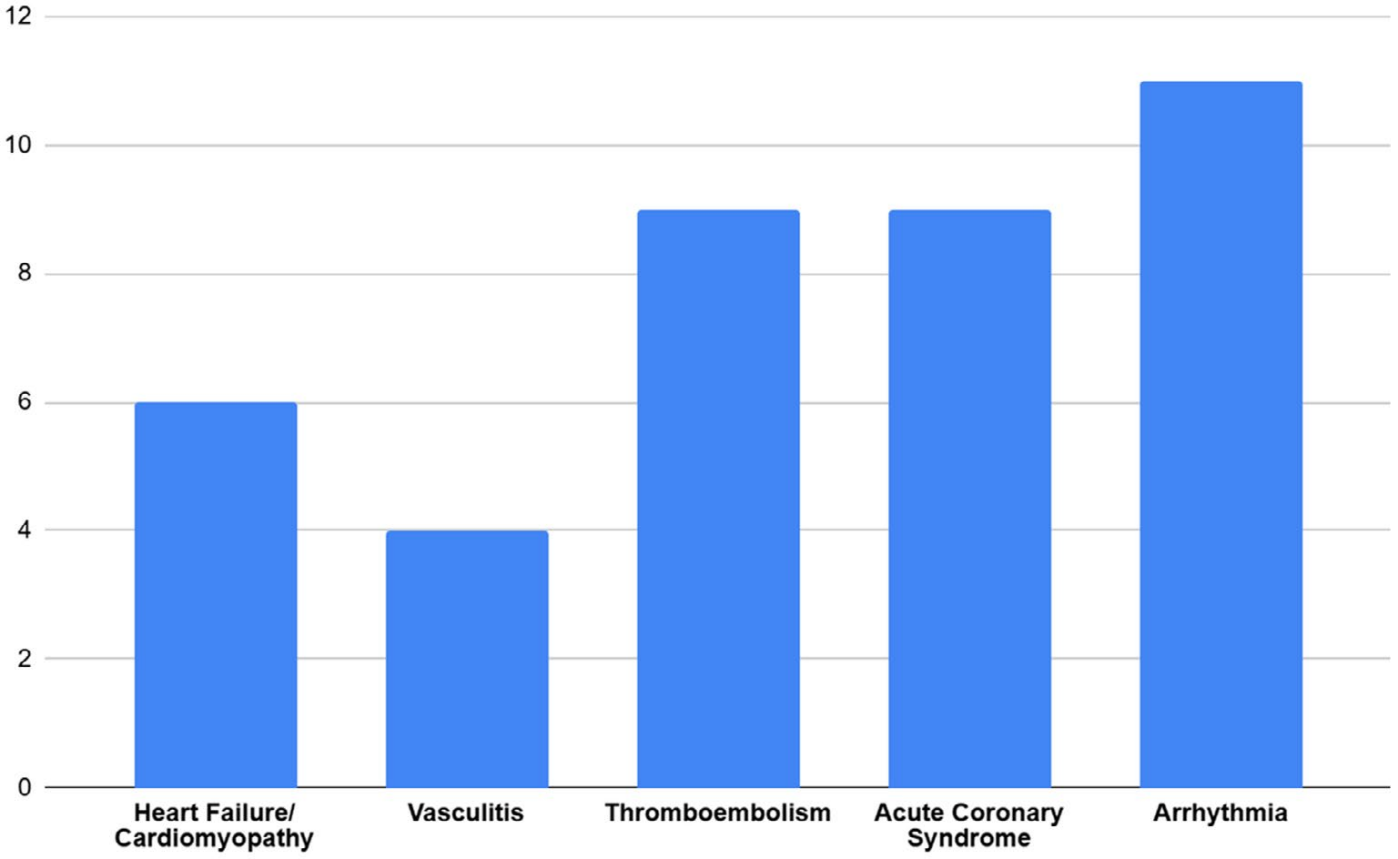
Distribution of total number of reported cardiovascular events by subtype.

The distribution of reported MACE by subtype is represented in Figure 2.

#### 3.4.1 Vasculitis

Four case studies described the onset of vasculitis after exposure to isotretinoin. These vasculitis events included three episodes of granulomatosis with polyangiitis, two of which were complicated by pulmonary–renal syndrome and the other by diffuse alveolar haemorrhage. There was one case of necrotising small‐vessel vasculitis of muscle.

#### 3.4.2 Thromboembolism

Nine case studies described thromboembolic events after exposure to isotretinoin, three of which caused acute coronary syndrome. The thromboembolic events included one pulmonary embolism, three cases of coronary thrombotic events, two thrombotic renal infarctions, one central vein occlusion and two cerebral ischaemic events.

#### 3.4.3 Cardiomyopathy

Two case studies described the onset of cardiomyopathy after exposure to isotretinoin. In the cohort study of matched controls, four cases of heart failure were described in the cohort exposed to isotretinoin; however, this was compared to 12 cases of new heart failure in the matched unexposed group.

#### 3.4.4 Arrhythmia

Five case studies described arrhythmias after exposure to isotretinoin, including two cases of atrial tachycardia, two cases of premature ventricular contraction and one case of a right bundle branch block. The matched cohort study described three cases of atrial fibrillation in the exposed group compared to 12 in the unexposed group, and three cases of ventricular tachycardia in the exposed group compared to four in the unexposed group.

#### 3.4.5 Acute Coronary Syndrome

Four case studies described episodes of acute coronary syndrome after exposure to isotretinoin, three of which were caused by thromboembolic events and one of which was caused by an allergic myocardial infarction. In the matched cohort study, there were five episodes of acute myocardial infarction in the exposed group compared to eight in the unexposed.

### 3.5 Morbidity and Mortality

Morbidity is also considered relating to ongoing medical issues after management of the acute MACE. Two reports described cases requiring ongoing medical management of heart failure; another detailed a case in which renal transplantation was required to manage the sequelae of vasculitis [32, 33]. One episode of granulomatosis with polyangiitis in a 14‐year‐old required long‐term immunosuppression with rituximab [25]. One case of cerebral venous thromboembolism in a previously healthy 27‐year‐old woman resulted in permanent hemiparesis [26].

There were no reported deaths from MACE in those exposed to isotretinoin.

## 4 Discussion

### 4.1 Strength of Evidence

This systematic review identified a paucity of high‐quality evidence for serious cardiovascular events in patients with acne who have taken isotretinoin. The highest quality evidence was a matched cohort study that showed no difference in MACE between those exposed to isotretinoin and unexposed matched controls [31]. This cohort included adults only but collected data over a 9‐year period of all patients with a diagnosis of acne attending a regional healthcare system in the United States. Data were collected on a large population of acne patients who were not exposed to isotretinoin, and analysis was performed on a propensity score–matched cohort to account for age, sex, comorbidities, baseline medications, socioeconomics and ethnicity. This created a robust cohort study for statistical analysis. Although the isotretinoin‐exposed group showed a lower number of MACE compared to the non‐exposed, there was no significant difference, likely due to the large matched cohort of almost 12,000 people in each group. Although this evidence is limited to adults and not as rigorous as a randomised controlled trial, it does provide strong evidence for the cardiac safety of acne patients treated with isotretinoin. Most of the remaining evidence consisted of case reports. Although the majority of case reports were of good quality, the overall level of evidence remains low. The causality of MACE due to exposure to isotretinoin could not be confirmed in any case report, according to the Naranjo Scale [8]. Laroche et al. (2007) described the only case deemed to have a ‘probable’ causality [23]. This report described the case of a 30‐year‐old man who developed recurrent cerebral ischaemia with clear unilateral neurological signs after 3 months on isotretinoin, on a background of having developed similar symptoms after exposure to isotretinoin 7 years prior. The cerebral ischaemia was confirmed on imaging, and his symptoms resolved 2 weeks after stopping isotretinoin. Despite the lack of causality in these case reports, the events described are serious and carry significant risk of morbidity and mortality. To further clarify this risk profile for potentially rare but serious adverse events, a consideration of possible underlying mechanisms is prudent.

### 4.2 Isotretinoin and the Inflammatory Cascade

The effects of isotretinoin on the inflammatory cascade involved in the pathophysiology of acne and isotretinoin‐related adverse events are complex. Insulin‐like growth factor‐1 (IGF‐1) is thought to be the main driver of inflammation in acne that is responsive to Isotretinoin [2]. IGF‐1 is upregulated in puberty and has been shown to be highly abundant in acne‐affected skin [34]. Further, congenital deficiencies of IGF‐1 resulted in an absence of acne, unless there was IGF‐1 supplementation [15]. IGF‐1 has also been shown to result in increased pro‐inflammatory cytokine production, specifically of NF‐KB, IL‐1, IL‐6, IL‐8 and TNF‐α [2]. When IGF‐1 binds to its receptor, through the phosphoinositide 3‐kinase/Akt pathway, it leads to an inactivation of pro‐apoptotic transcription factors forkhead box class O (Fox) O1 and O3 and an inhibition of the transcription factor p53 [35]. Specifically, p53 is needed to maintain baseline FoxO1 expression, and the suppression of FoxO1 is thought to underpin acne formation as it allows lipogenic and pro‐inflammatory genes to increase sebaceous lipogenesis [16]. Isotretinoin acts on multiple sites in the signalling cascade via its metabolites to increase FoxO1, FoxO3 and p53, thus increasing apoptosis of sebocytes and decreasing sebum production [34, 36]. This is also supported immunohistochemically with a study that demonstrated increased p53 expression after isotretinoin treatment compared to that before commencing treatment and controls [34]. However, it also accounts for isotretinoin's many adverse effects such as apoptosis of neural cells (teratogenicity), granulosa cells (reduction in ovarian reserve) and hypothalamic neurons (depression) and, of relevance to this systematic review, hyperlipidaemia [37].

### 4.3 Isotretinoin and Lipids

Our consideration of the possible cardiovascular risk from the use of isotretinoin in acne patients stemmed from the well‐documented isotretinoin‐induced elevation of low‐density lipoprotein (LDL) in this population [38]. This effect was documented in a prospective cohort study that documented a clear elevation of LDL and a reduction in high‐density lipoprotein (HDL) compared to baseline after treatment with isotretinoin [32]. Karapinar, Polat, and Buğdaycı (2020) clarified this effect to show that both LDL and LDL receptors were upregulated during exposure to isotretinoin [39]. Interestingly, this same pattern was recognised by Georgala et al. (1997); however, they also concluded that the use of isotretinoin significantly reduced levels of lipoprotein‐a despite an increase in LDL [40]. It was suggested that this reduction in lipoprotein‐a may balance the cardiovascular risk from elevated LDL. Neele et al. (2001) found no change in lipoprotein‐a levels in 10 male patients without acne being treated with isotretinoin for 5 days [41]. In contrast, Dursun et al. (2011) found an increase in lipoprotein‐a levels after the treatment of 45 acne patients with isotretinoin for 6 months [42]. These contradictory results may represent differences in background genetic profiles of studied groups and may be independent of isotretinoin treatment. Vinci et al. (2023) report the difficulties in characterising the role of lipoprotein‐a in cardiovascular disease due to large inter‐individual genetic variation within the population [43]. It has been reported as an individual risk factor for cardiovascular diseaseand is unable to be targeted by standard lipid‐lowering therapies. Larger studies are required to measure lipoprotein‐a levels in acne patients compared to those without acne and also to measure the levels before and after extended isotretinoin treatment in both groups to clarify these current contradictory results.

While this relationship remains unclear, Melnik (2010) hypothesised that the mechanism underlying hypercholesterolaemia secondary to isotretinoin is related to the upregulation of p53 and FoxO transcription factors from binding to retinoid receptors [36]. The upregulation of p53 and FoxO1 transcription factors has a multitude of downstream effects, including increasing the expression of lipoproteins. Furthermore, FoxO1 also increases the expression of the *APOB* gene that increases apolipoprotein B‐100 (apoB100) production, which is necessary for the assembly and secretion of VLDLs from the liver, and the *APOC3* gene that increases the production of apolipoprotein C‐III [44]. Apolipoprotein C‐III inhibits the uptake and metabolism of lipoproteins, and so its increased expression due to isotretinoin reduces the regulation of LDL, enabling high levels to accumulate. Therefore, collectively, isotretinoin results in increased triglycerides via supporting VLDL assembly and secretion while reducing the uptake of VLDLs and IDLs [37].

### 4.4 Isotretinoin and Thromboses

Case reports were identified drawing a link between thromboembolic events and isotretinoin treatment in acne patients. In the nine case studies describing thromboembolic events, only one case described the concomitant use of the combined oral contraceptive pill. The possible mechanisms underlying this association are again complex; however, they may be connected to the elevation of lipids via the upregulation of p53 and Fox transcription factors described above [45]. Upregulation of these factors has been shown to increase the expression of lipoprotein(a). Elevation of lipoprotein(a) has been associated with impairment of thrombolysis by inhibiting the activity of plasminogen and increased generation of thrombi via enhanced platelet aggregation [37, 40]. Furthermore, isotretinoin has been shown to upregulate caspase 1, which activates a pro‐inflammatory cytokine into interleukin‐1 (IL‐1) [37]. This activation of IL‐1 has been associated with a prothrombotic action [46].

### 4.5 Isotretinoin and Inflammation

Isotretinoin has been shown to have a complex effect on multiple inflammatory pathways [37]. The activation of IL‐1, as described above, has been implicated as a potential cause of the initial inflammatory effect underlying acne fulminans when starting isotretinoin treatment for acne [37]. Furthermore, the inflammatory effect of IL‐1 has also been associated with the development of cardiac hypertrophy and subsequent heart failure [47]. This pro‐inflammatory effect of isotretinoin through IL‐1 therefore may provide a mechanism for the onset of MACE due to the development of cardiac hypertrophy.

### 4.6 Isotretinoin and Cardiac Remodelling

Soriano et al. (2013) assessed change in structural cardiac measurements due to isotretinoin [48]. A total of 20 males with acne were treated with isotretinoin and had Doppler echocardiogram readings at baseline and after 10 weeks of treatment. This study found a significant increase in interventricular septal thickness and left ventricular mass and decreased left ventricular cavity size and cardiac index. These measurements are suggestive of increased cardiac hypertrophy and myocardial structural remodelling due to isotretinoin. Zhong et al. (2004), however, suggest that these echocardiographic changes due to isotretinoin may not reflect structural change and remodelling [49]. Instead, they suggest that these changes may be due to a vasodilatory effect of isotretinoin via increased expression of angiotensin‐converting enzyme‐2. Although there is a possible isotretinoin‐induced inflammatory cause for cardiac hypertrophy, the findings described in this evidence may also be due to a temporary vasodilatory effect. The evidence for significant cardiac structural remodelling from isotretinoin remains uncertain; however, it may explain possible mechanisms underlying the onset of rare MACE.

### 4.7 Isotretinoin and Arrhythmia

The results of this systematic review described the possible onset of arrhythmias secondary to isotretinoin. A study of 26 acne patients treated with isotretinoin for 3 months found no arrhythmias on 24‐h Holter monitoring [50]. Dursun et al. (2011) further investigated possible alterations to cardiac conduction during treatment with isotretinoin, as a possible cause for associated arrhythmias [19]. They investigated 45 patients with acne, on 0.8 mg/kg/day of isotretinoin for 6 months, and found no significant change in QT or QT dispersion. Furthermore, Ay, Aksoy, and Güngören (2019) found no difference in QT or QT dispersion after 1 month of isotretinoin in 30 patients [51]. These studies have small sample sizes and may not be adequately powered; hence, the evidence for alteration to cardiac conduction due to isotretinoin remains unclear. To further clarify the cardiovascular risk of exposure to isotretinoin, a consideration of potential confounders is required.

### 4.8 Review of Confounders

Although serious cardiovascular events during isotretinoin treatment, as described in the case reports of this systematic review, may be explained by several aforementioned underlying pathophysiological mechanisms, it is necessary to consider possible confounders. Generally, the patients with acne treated with isotretinoin are younger adults. It is possible that other risk factors for thromboembolic events were present but undetected. Similarly, the detection of arrhythmias and heart failure could potentially be incidental or unmasked at the time of isotretinoin treatment. It is possible that structural abnormalities or cardiomyopathies could have underlying genetic backgrounds or be associated with other environmental factors. From another perspective, it is possible that these events could be related to severe auto‐inflammatory cascades relating to the underlying acne rather than exposure to isotretinoin.

### 4.9 Limitations

The major limitation of this systematic review is the low‐evidence‐level case reports that were included and the lack of high‐quality randomised‐controlled trials. This reduces the generalisability of results and the quality of evidence and limits the ability to conduct rigorous statistical analysis. The reliance on evidence from case reports must be considered with a level of selection bias in these cases that may not be generalisable to all acne patients taking isotretinoin. Observer bias must also be considered in the assessment of each author's determination of the link between isotretinoin treatment and the development of MACE.

### 4.10 Recommendations

This review does not find any significant relationship between the onset of MACE in patients with acne who receive treatment with isotretinoin. Given the uncertain data on the effects of isotretinoin on the independent cardiovascular risk factor of lipoprotein‐a levels in acne patients, and the serious nature of the events described, it is reasonable to consider cardiovascular risk in acne patients on isotretinoin. Cardiovascular history and cardiac risk factors should be considered prior to treatment, including a baseline lipid profile including LDL, HDL and triglycerides. Further screening of lipid profiles should be repeated if cardiovascular events occur and could be considered at the end of treatment to ensure resolution of isotretinoin‐induced alterations. If cardiovascular events occur during treatment with isotretinoin, stopping treatment and assessing the relationship with isotretinoin should be conducted on an individual basis and accounting for other possible causes.

## 5 Conclusion

Ultimately, this systematic review found weak evidence for causality of MACE secondary to isotretinoin use for the treatment of acne, despite a well‐documented association with hypercholesterolaemia. The evidence of the occurrence of MACE during isotretinoin treatment for acne is largely confined to case reports that do not confirm causality. Due to this lack of certainty, it is essential to consider other possible confounders that might contribute to the onset of MACE in this patient population. Given the morbidity and mortality risk from MACE, however, further consideration of potential underlying mechanisms that may confer this risk is suggested. Furthermore, educating patients to seek medical attention for any symptoms of MACE could be recommended, given the occurrence of rare events.

## Author Contributions

T.L.C. and A.M. were involved in all components of study design, analysis, data interpretation and manuscript production. M.S. was involved in study analysis; data extraction and interpretation; and manuscript drafting, editing and approval. S.S. was involved in data analysis; interpretation; manuscript drafting, editing and final approval.

## Disclosure

AI was not used in preparation of this manuscript.

## Ethics Statement

The authors have nothing to report.

## Conflicts of Interest

The authors declare no conflicts of interest.

## Data Availability

The data that support the findings of this study are available from the corresponding author upon reasonable request.

## Appendix: Search Terms A

**Embase, Scopus, Pubmed 7/11/2023**

**Table:**

	Search term	Results
1	acne.ti,ab.	26076
2	isotretinoin/	14507
3	(isotretinoin or "13‐cis‐retinoic acid" or "13 cis retinoic acid" or "13‐cis‐isomer" or "zn salt" or eh28up18if or "13 cis isomer" or "ro 4 3780" or "vitamin A").ti,ab.	32966
4	(A‐Cnotren, or Absorica or Accuran or Accutane or Accutin or Acnecutan or Acnegen, or Acnemin or Acneone or Acneral or Acnestar or Acnetane or Acnetin A or Acnetrait or Acnetrex or Acnogen or Acnotin or Acnotren or Acretin or Actaven or Acugen or Acutret or Acutrex or Ai Si Jie or Aisoskin or Aknal or Aknefug Iso or Aknenormin or Aknesil or Aknetrent or Amnesteem or Atlacne or APO‐Isotretinoin or Atretin or Axotret or Casius or Ciscutan or Claravis or Contracne or Curacne or Curakne or Curatane or Cuticilin or Decutan or Dercutane or Dermatane or Effederm or Epuris or Eudyna or Farmacne or Flexresan or Flitrion or Inerta or Inflader or Inotrin or Isac or Isdiben or Isoacne or Isobest or Isocural or Isoderm or Isoface or IsoGalen or Isogeril or Isolve or Isoprotil or Isoriac or Isosupra or Isosupra or Lidose or Isotane or Isotina or Isotinon or Isotren or Isotret or lsotretinoin or Isotretinoina or Isotretinoine or Isotretinoine or Isotretinoinum or Isotrex or Isotrin or Isotroin or Izotek or Izotziaja or Lisacne or Locatret or Mayesta or Myorisan or Neotrex or Netlook or Nimegen or Noitron or Noroseptan or Novacne or Oralne or Oraret or Oratane or Piplex or Policano or Procuta or Reducar or Retacnyl or Retin A or Roaccutan or Roaccutane or Roacnetan or Roacta or Roacutan or Rocne or Rocta or Sotret or Stiefotrex or Tai Er Si or Teweisi or Tretin or Tretinac or Tretinex or Tretiva or Tufacne or Zenatane or Zerocutan or Zonatian or Zoretanin).ti,ab,tn.	3662
5	or/2‐4	42499
6	exp cardiovascular disease/	4836864
7	(heart* or cardio* or cardiac or myocard* or pericard* or atrial or ventricular or arrythmia or fibrillation or dysrhythmia or syncop* or stroke* or clot* or thrombos* or embol* or thromboembol* or isch?em* or atheroscler* or hyperension).ti,ab.	4097090
8	6 or 7	6091170
9	1 and 5 and 8	419

**Medline**

**Table:**

	Search term	Results
1	exp Acneiform Eruptions/	13906
2	acne.ti,ab.	16048
3	1 or 2	19166
4	Isotretinoin/	3990
5	(istretinoin or "13‐cis‐retinoic acid" or "13 cis retinoic acid" or "13‐cis‐isomer" or "zn salt" or eh28up18if or "13 cis isomer" or "ro 4 3780" or "vitamin A").ti,ab.	25451
6	(A‐Cnotren, or Absorica or Accuran or Accutane or Accutin or Acnecutan or Acnegen, or Acnemin or Acneone or Acneral or Acnestar or Acnetane or Acnetin A or Acnetrait or Acnetrex or Acnogen or Acnotin or Acnotren or Acretin or Actaven or Acugen or Acutret or Acutrex or Ai Si Jie or Aisoskin or Aknal or Aknefug Iso or Aknenormin or Aknesil or Aknetrent or Amnesteem or Atlacne or APO‐Isotretinoin or Atretin or Axotret).ti,ab,kf.	198
7	(Casius or Ciscutan or Claravis or Contracne or Curacne or Curakne or Curatane or Cuticilin or Decutan or Dercutane or Dermatane or Effederm or Epuris or Eudyna or Farmacne or Flexresan or Flitrion).ti,ab,kf.	17
8	(Inerta or Inflader or Inotrin or Isac or Isdiben or Isoacne or Isobest or Isocural or Isoderm or Isoface or IsoGalen or Isogeril or Isolve or Isoprotil or Isoriac or Isosupra or Isosupra).ti,ab,kf.	328
9	(Lidose or Isotane or Isotina or Isotinon or Isotren or Isotret).ti,ab,kf.	12
10	(Isotretinoin or Isotretinoina or Isotretinoine or Isotretinoine or Isotretinoinum or Isotrex or Isotrin or Isotroin or Izotek or Izotziaja or Lisacne or Locatret).ti,ab,kf.	3366
11	(Mayesta or Myorisan or Neotrex or Netlook or Nimegen or Noitron or Noroseptan or Novacne or Oralne or Oraret or Oratane or Piplex or Policano or Procuta or Reducar or Retacnyl or Retin A or Roaccutan or Roaccutane or Roacnetan or Roacta or Roacutan or Rocne or Rocta or Sotret or Stiefotrex or Tai Er Si or Teweisi or Tretin or Tretinac or Tretinex or Tretiva or Tufacne or Zenatane or Zerocutan or Zonatian or Zoretanin).ti,ab,kf.	167
12	or/4‐6	28458
13	exp Cardiovascular Diseases/	2713940
14	(heart* or cardio* or cardiac or myocard* or pericard* or atrial or ventricular or arrythmia or fibrillation or dysrhythmia or syncop* or stroke* or clot* or thrombos* or embol* or thromboembol* or isch?em* or atheroscler* or hyperension).ti,ab.	2676803
15	13 or 14	3768621
16	2 and 12 and 15	59
